# Pop-rock musicians: Assessment of their satisfaction provided by hearing protectors

**DOI:** 10.1590/S1808-86942010000400009

**Published:** 2015-10-19

**Authors:** Cristiane Bolzachini Santoni, Ana Claudia Fiorini

**Affiliations:** a Master's degree in speech therapy, Pontfcal Catholic University, Sao Paulo (PUC/SP). Speech therapist; b Doctoral degree in public health, FSP/USP. Speech therapist, associate professor at the PUC/SP

**Keywords:** hearing, ear protective devices, music, hearing loss

## Abstract

Pop-rock musicians are at risk of developing hearing loss and other symptoms related to amplified music.

**Aim:**

The aim of the present study was to assess the satisfaction provided by the use of hearing protection in pop-rock musicians. Study design: Contemporary cohort study.

**Materials and Methods:**

A study of 23 male pop-rock musicians, aged between 25 to 45 years. After audiological evaluation (pure tone audiometry, middle ear analysis, TEOAE and DPOAE) hearing protective devices were provided to be used for three months. After that musicians answered a satisfaction assessment questionnaire.

**Results:**

The prevalence of hearing loss was of 21.7%. The most common complaints about the hearing protectors were: autophonia, pressure in the ears, interference in high frequencies perception and full time use of the hearing protector during concerts. There was a positive correlation between a reduction in tinnitus after the use of the HPD with the following complaints: tinnitus after beginning the career (p= 0.044), discomfort with the sound intensity in the work place (p= 0.009) and intolerance to loud sound (p= 0.029).

**Conclusions:**

There was a high prevalence of hearing loss and a positive tendency towards the use of the ear protector device among the sample population.

## INTRODUCTION

Since the 1960s several authors have asked whether sound levels from amplification systems used by pop rock and rock and roll bands are harmful to hearing and could permanently damage the auditory apparatus. Studies haves hown that the prevalence of hearing loss among musicians ranges from 5% to 52%.^1^, ^2^, ^3^, ^4^, ^5^, ^6^

Aside from the auditory effects that electronically amplified music may cause, other studies have reported non-auditory findings that affect the quality of life of music professionals. These include tinnitus, dizziness, hyperacusis, sound distortion, fullness in the ear, altered cardiovascular, gastric and muscular systems, changes in humor, stress, and irritability.^4^^,^^7^, ^8^, ^9^, ^10^ Preventive measures could be: acoustic treatment of the presentation ambience, audiological monitoring with pure tone audiometry and otoacoustic emissions, and hearing protector aids.

The main feature of hearing protectors for musicians is to have uniform attenuation, never attenuating more the higher frequencies relative to middle and low frequencies, as happens with common hearing protectors. They are named high fidelity hearing protectors because the original quality of music is preserved, but at a lower sound level. Custom models may reduce sound by 9dB, ^15^ dB or 25 dB, depending on the filter. The choice is based on the type of exposure to noise. Premolded protectors may provide a 20 dB sound attenuation.^11^, ^12^, ^13^

A study showed that musicians who used hearing protectors assiduously were those that presented some hearing complaints, comprising about 20% of a sample of 196 musicians. They had started using hearing protectors after perceiving initial hearing symptoms of any type. The most common types used in this group were custom insert (47%) and premolded (25%) hearing protectors. The author concluded that the presence of hearing complaints could positively affect the decision of using a hearing protector.^14^

A survey of young pop-rock musicians assessed the acceptance of musician-specific hearing protectors. Although these protectors were specifically made for musicians, a few sound quality issues were noted, such as occlusion effects and altered perception of high frequencies. There was a negative trend relative to the use of hearing protectors throughout shows. Nevertheless, a qualitative analysis of hearing protectors generally revealed a positive receptivity among musicians.^15^

In this context, it is necessary to assess the effects that exposure to amplified music generate to health in general and hearing in these professionals, and to prevent such losses by the use of individual hearing protectors that do not distort music quality.

The purpose of this study was to assess the satisfaction level of hearing protector use among pop rock bands.

## MATERIALS AND METHODS

This study consisted of a contemporary cohort investigation of pop-rock musicians from June to November 2007.

Prior to the study, participants were informed about the procedures and signed a free informed consent form. The institutional review board of the institution approved the study (numbers 009/2007 and 002/2007).

Fifty musicians belonging to several pop rock bands were invited to participate. Only 24 of them accepted the invitation and underwent the study procedures. Others stated that they were interested, but presented the following reasons for not participating: fear of discovering hearing loss; too many commitments; a full agenda; lack of interest or rejection about using hearing protectors. Similar difficulties have been reported in other studies of musicians.^1^^,^^3^^,^^16^

Subjects with any of the following were excluded from the sample: age over 50 years; presence of conductive or mixed hearing loss; altered tympanometric curve in acoustic immittance testing; and presence of preexisting diseases and other neurological or degenerative diseases. Thus, of 24 subjects, one was excluded because of a left type B tympanometric curve.

The study sample therefore consisted of 23 male subjects who had been musicians of pop rock bands for at least one year. These bands comprised 3 to 5 members, and each participant was characterized according to his or musical instrument, as follows: voice (8), electric guitar (7), bass guitar (4), double bass (4), guitar (4), drums (4), and keyboards (1). It should be noted that some of them played more than one musical instrument.

Ages ranged from 25 to 45 years; the mean age was 32.4 years, and the standard deviation was 4.5 years. The time spent at work ranged from 3 to 21 years; the mean was 13.4 years, and the standard deviation was 4.7 years. The exposure time to sound in hours per week ranged from 1.5 to 20 hours; the mean was 10 hours per week, and the standard deviation was 5.5 hours per week. Thus, the sample mostly comprised musicians aged from 25 to 35 years (82.6%), who had spent from 6 to 15 years working (78.3%), and who were exposed to music during shows from 6 to 10 hours per week (47.8%).

After passing the selection criteria, subjects answered a questionnaire adapted from those applied in previous studies.^9^^,^^16^^,^^17^ The following information was gathered: personal data on current and previous general health, exposure to amplified music, hearing and non-hearing complaints, perceptions of hearing findings after work and other variables that might affect the results of audiological tests.

Visual inspection of the outer ear canal was done before audiological testing to check for any obstruction that might preclude further testing. If this was the case, these subjects were excluded from the study and referred for an otorhinolaryngological evaluation.

Next, the following hearing tests were applied: pure tone audiometry, acoustic immittance testing, transient-evoked otoacoustic emissions testing (TEOAE) and distortion product otoacoustic emissions testing (DPOAE). An auditory rest of at least 14 hours was given between tests.^18^

After audiological testing, each musician was given a pair of E.A.R. ER 20 high-fidelity hearing protectors, which is a premolded single size silicone three-flange earplug. The manufacturer informs that this hearing protector provides a linear sound level frequency decrease and does not distort voice or music. Its noise reduction rating is 12 dB.^19^

The reasons for choosing this hearing protector were: uniform attenuation preserving the fidelity of the original sound signal; a single size not requiring experts to make a mold; lower cost, and adequate attenuation for the needs of the study group.^20^

Musicians were given instructions about the placement and hygiene of the hearing protector. They were asked to use the hearing protectors in their shows for the next three months.^15^^,^^20^

After this period they were asked to fill in a standard questionnaire^15^ without researcher input to assess the use and acceptance of the hearing protector and gauge user satisfaction. The questionnaire consisted of questions about determining factors for hearing protector use, such as: quality of sound perception, and comfort and ease of use. For each statement subjects were asked to mark an agreement/disagreement answer (“agree fully,” “agree,” “indifferent,” “disagree,” and “disagree completely”). These answers were converted into a numerical code for a five to one score. The scores of each statement were added, where the minimum value was 10 points (negative attitude) and the maximum value was 50 points (positive attitude). Negative sensations while using the hearing protector were surveyed, as was the perception of decreased hearing complaints; a score was given to express the degree of satisfaction with using the hearing protector.

The SPSS (Statistical Package for the Social Sciences) version 13.0 software was used for the statistical analysis. The Mann-Whitney test was applied for studying the parametric values. The chi-square and Fisher's exact test were applied to study non-parametric variables. The significance level throughout was 5% (α=0.050).

## RESULTS

The most frequent hearing complaints of musicians upon entering their profession and just after a show were tinnitus (39.1% upon entering the profession, and 56.5% after a show) and intolerance to loud sounds (34.8% upon entering the profession and 30.4% after a show). The most common extra-auditory complaints were insomnia (26.1%) and memory problems (26.1%).

Pure tone audiometry revealed that the highest mean audiometric threshold values occurred at 3000, 4000 and 6000 Hz in both ears. Classifying the audiograms based on Fiorini (1994)^21^ showed that 78.3% were within normal limits and 21.7% had tracings that suggested noise-induced hearing loss (NIHL). Although most of the audiograms were within normal limits, there were many unilateral or bilateral notches (56,5%), mostly at 6000 Hz.

TEOAE revealed a higher occurrence of positive responses bilaterally (52.2%). The sum of unilateral and bilateral absent responses comprised 47.8% of subjects, similar to one ear only. DPOAE showed absent responses in 56.5% of cases, especially in the bilateral condition (34.8%).

Table 1 shows the distribution of responses, the mean, and the standard deviation for each statement on the degree of agreement when expressing satisfaction with using the HiFi ER 20 hearing protector. In most statements, the tendency of responses was more frequent for the intervals “agree fully” and “agree.” Note that the response trend was positive, especially about the following aspects: the hearing protector allows one to hear other instruments of the band with quality, it allows one to identify the timbre of other instruments, it allows one to perceive bass sounds, and to hear the vocalist clearly. The mean total score, which ranged from 10 to 50, was 36.2.

**Table 1:** Distribution of responses relative to the degree of agreement with each statement in the questionnaire on satisfaction when using the HiFi ER 20 hearing protector (n=23).

STATEMENT	Mean*	Standard Deviation				Degree of Agreement						
The ER 20 hearing protector (…)			CT		C		I		D		DT	
			N	%	N	%	N	%	N	%	N	%
(…) possible to hear all other band instruments with quality.	3,7	1,0	5	21,7	11	47,8	3	13,0	4	17,4	–	–
(…) possible to identify the timbre of other instruments.	4,0	0,6	4	17,4	15	65,2	4	17,4	–	–	–	–
(…)possible to clearly perceive treble sounds.	3,1	1,2	2	8,7	10	43,5	2	8,7	7	30,4	2	8,7
(…)possible to clearly perceive bass sounds.	3,9	0,9	5	21,7	15	65,3	1	4,3	1	4,3	1	4,3
(…)possible to clearly hear the vocalist.	3,7	1,2	7	30,4	8	34,8	4	17,4	2	8,7	2	8,7
(…) it is comfortable/snug	3,3	1,2	4	17,4	6	26,1	8	34,8	3	13,0	2	8,7
Interference of the ER 20 hearing protector on esthetics of musician is negligible	3,4	1,2	5	21,7	6	26,1	5	21,7	6	26,1	1	4,3
(…) easy to place	4,4	0,9	15	65,3	4	17,4	2	8,7	2	8,7	–	–
I would use a hearing protector like this full time during rehearsals	3,6	1,5	10	43,5	5	21,7	1	4,3	4	17,4	3	13,0
I would use a hearing protector like this full time during shows.	2,9	1,5	4	17,4	6	26,1	3	13,0	4	17,4	6	26,1
TOTAL MEAN SCORE	36,2	6,2										

Key: CT: agree fully; C: agree; I: indifferent; D: disagree; DT: disagree fully.

* mean and standard deviation: the maximum score for each statement was 5 points, and the total score ranged from 10 to 50 points.

Of 23 musicians, 18 (78.3%) scored over 30 points, showing that the hearing protector satisfactorily met user expectations and needs. When asked about the score (from 0 to 10) given to demonstrate satisfaction with using the hearing protector, six musicians (26.1%) scored 6 or less, seven musicians (30.4%) score 7, and ten musicians (43.5%) scores 8 and 9. Thus, 73.9% of scores were between 7 and 9, which reveals a positive trend in favor of satisfaction with the use of the HiFi ER 20 hearing protector.

Questionnaire statements were divided into three categories:15 quality of sound reception, comfort, and ease of use. The mean values resulted from the sum of scores for each agreement/disagreement intervals indicated by musicians (from 0 to 5).

The fist category concerned the quality of sound reception, where the mean values were: quality perception of band instruments (3.7), perception of the timbre of band instruments (4.0), perception of high frequency sounds (3.1), perception of low frequency sounds (3.9), and clear perception of the vocalist's voice (3.7). The lowest mean values were found in the statement about interference in perceiving high frequency sounds.

The second category was related to comfort when using the hearing protector, where the mean value was 3.3 for comfort while using the protector and 3.4 for its esthetics.

The third category, ease of use, yielded the following mean values: ease of placing the hearing protector (4.4), using the protector full time during rehearsals (3.6), and using the protector full time during shows (2.9).

The most frequent negative sensations while using the hearing protector were dampened voice (43.5%), and pressure on the ears (39.1%) (Table 2). The occurrence of three or more negative sensations associated with hearing protector use was the most frequent (43.5%). Only 4.3% of musicians reported not perceiving any negative sensation while using the hearing protector.

**Table 2:** Distribution of negative sensations reported by musicians while using the HiFi ER 20 hearing protector (n=23).

Negative Sensations	Number	Percentage
Feeling dampened voice	10	43,5%
Pressure in ears	9	39,1%
Pain in the ear	7	30,4%
Difficulty with music return	7	30,4%
Feeling isolated	6	26,1%
Ear itching	6	26,1%
Interference with music quality	6	26,1%
Communication difficulties	5	21,7%
Feeling of blocked ear	5	21,7%
Ear warmth	2	8,7%
Protector fell from ear	2	8,7%
Mild discomfort	1	4,3%

Fischer's exact test found positive correlations between the perception of decreased tinnitus after using the HiFi ER 20 hearing protector and the following variables: presence of a complaint of tinnitus after entering the profession (p=0.044), reports of feeling bothered with the sound level at the worksite (p= 0.009), and complaints of intolerance to loud sounds (p= 0.029).

The Mann-Whitney test related hearing complaints with satisfaction indicators with use of the HiFi ER 20 hearing protector; the results were statistically significant for the following conditions:

• Presence of a complaint of tinnitus and sensation of the HiFi ER 20 hearing protector interfering with the quality of music (p=0.028),
• Presence of tinnitus and a sensation of decreased tinnitus after using the hearing protector (p=0.031),
• Presence of a complaint of intolerance to loud sounds and a sensation of decreased tinnitus after using the hearing protector (p=0.015),
• Presence of feeling bothered with the sound intensity to which one is exposed, and an assessment that the hearing protector had a positive influence on performance (p=0.042),
• Presence of feeling bothered with the sound intensity to which one is exposed and the sensation of decreased tinnitus after using the hearing protector (p=0.006), and
• Prior use of any type of hearing protector and an assessment that the protection provided by the HiFi ER 20 model was satisfactory (p=0.041).

## DISCUSSION

The main hearing complaints reported by pop-rock musicians in this study were tinnitus (39.1%) and intolerance to loud sound (34.8%). These findings corroborate several national and international studies that have confirmed these problems as the most frequent in musicians. In these studies, the frequency of the complaint tinnitus ranged from 37.5% to 45%, and the complaint intolerance to loud sound ranged from 19% to 48%.^4^^,^^6^, ^7^, ^8^^,^^14^^,^^16^^,^^22^, ^23^, ^24^, ^25^

The same complaints were also reported after shows; tinnitus in this situation may be related to a temporary change in hearing thresholds as a result of exposure to highly amplified music, even for short time periods. It should be noted that the average daily exposure was three hours and the average weekly exposure was ten hours, which are lower than a regular workday (8h/day or 40h/ week). The weekly average exposure time was similar to that in other studies.^3^^,^^15^^,^^16^

Early studies in 1967 and 1974 on the prevalence of hearing loss in musicians showed rates of around 8%.^1^ Over the years, however, studies have reported variations in this rate: 22%,^1^ 23%,^22^ 33%,^3^ 8%,^4^ and 50% in chronological order.^26^ The variation in prevalence could be due to different audiogram classification criteria. Note that increased hearing loss in musicians would be expected when we consider that technology has made possible considerably increased sound levels during shows. The 21.7% hearing loss prevalence at high frequencies (from 3000 to 6000 Hz) encountered in this study is within the mean rates of other studies.

Also note that this prevalence of NIHL-suggesting audiograms is similar to that in steel mills, notwithstanding exposure to different sound sources and time spent in the noisy environment.^21^ Such similarity is cautionary, coming from a different reality where workers generally spend eight daily hours at work compared to three daily hours of exposure for musicians. It may thus be assumed that musicians are at a higher risk for hearing loss, even with a shorter daily exposure time (daily or weekly hours, compared to factory workers. There is both the fact that musicians are exposed to higher noise levels for short time periods and that Hearing Loss Prevention Programs (HLPP) are scarce in this population.

Absent otoacoustic emissions test responses (OAE) were frequent in this study; 78.3% of musicians had audiometric thresholds within normal limits. This result may reflect the fact that OAE are a measurement of cochlear motor activity due to outer hair cell action. Because hearing loss caused by high sound pressure levels starts with outer hair cell damage,^27^, ^28^, ^29^, ^30^, ^31^ OAE may be more sensitive than pure tone audiometry to detect early changes due to exposure to amplified music.^4^^,^^7^^,^^16^^,^^25^^,^^26^^,^^31^

Responses in the questionnaire on satisfaction with the HiFi ER 20 hearing protector were concentrated in the “agree” and “fully agree” groups, indicating a positive attitude towards using hearing protectors. Two questions were exceptions: clear perception of high frequency sounds, and full-time use of the ER 20 hearing protector during shows. For the first question, 39.1% “disagreed” and “strongly disagreed,” suggesting that, even with uniform attenuation, the hearing protector alters the perception of high-frequency sounds. For the latter question, 43.5% of responses to full-time use of hearing protectors during shows were “disagree” and “strongly disagree,” which indicates resistance to hearing protector use.

A study of young pop-rock musicians has also found that the worst evaluation scores refer to similar aspects.^15^ The author stressed that altered perception of high frequency sounds has a relevant influence on the perception of sound quality. Although musicians recognize the need for hearing protection, they rarely accept this type of altered frequency perception, which affects the clarity of musical instruments.

Musicians that had no interest in using the hearing protector full time during shows commented in the questionnaire that compliance was low for specific performance reasons, namely that there was interference with sound fidelity and referential normal hearing. These findings were similar to those observed in a study of instrumental band musicians; it assessed compliance with the same type of hearing protector.^20^ A study of Danish symphony orchestra musicians revealed that contributing factors against the use of hearing protectors were similar to the above, namely difficulty hearing other instruments and interference with their own performance.^31^

The most important point favoring the use of hearing protectors in a study of opera and classical musicians was perception of the sonority of the instrument itself, that is, whether use of hearing protectors did or did not generate sound distortion of instruments.^32^

The most frequent negative sensations due to protectors were dampened voice (43.5%) and pressure in the ear (39.1%). Only 4.3% of musicians said they had no negative sensations with protectors.

Some authors have attributed sensations of pressure and dampened voice to an occlusion effect.^13^^,^^31^^,^^33^ These sensations may be so uncomfortable as to result in musicians not using hearing protectors.^31^

Pressure in the ears may also be explained by the relative size of the hearing protector.^34^ A single size protector was used in this study, which may have resulting in this sensation because of size variations of outer ear canals; another study of pop rock musicians had a similar finding.^15^ Discomfort may be reduced with continued use of hearing protectors.

Negative feelings observed in this study were similar to those in a study of workers exposed to occupational noise designed to assess comfort with hearing protector use. The authors reported that interference with comfort was due to difficulties in conversation (53.4%) and pressure in the ears (39.4%). Only 5% of the sample reported absence of discomfort.^35^

Our findings differed from those of a study of orchestra musicians^14^ where the most frequently reported negative feeling was that hearing protectors affected performance and made it difficult to hear other instruments, thereby affecting usual auditory references.

A significant positive correlation was found between hearing complaints (tinnitus, annoyance with noise exposure and intolerance to loud sounds) and perception of decreased sensation of tinnitus after using hearing protectors. It is known that tinnitus is among the effects of exposure to excessive sound pressure levels, annoyance with exposure to noise and intolerance to loud sounds. Exposure levels are attenuated when using hearing protectors; it is therefore possible to perceive its benefits.^4^^,^^5^^,^^7^^,^^13^

A relation between the presence of hearing complaints (tinnitus and intolerance to loud sound) and variables considered as indicators of the degree of satisfaction with hearing protectors was found. The results corroborate research findings showing that hearing complaints interfere in the evaluation and use of hearing protectors.^14^^,^^31^^,^^32^

According to survey data, more hearing complaints relate to increased concern by musicians about their hearing; they are consequently more aware of the perception of discomfort with noise exposure at work. In such cases there is more acceptance of hearing protectors and its use in rehearsals and performances becomes more frequent.^31^

After using hearing protectors for three months, 73.9% of the sample gave a score over 7.0 for satisfaction with use. Furthermore, 78.3% of the questionnaires had scores above 30 points. These findings suggest that hearing protectors satisfactorily met the needs and expectations of users and indicate a positive trend for accepting hearing protectors.

The overall results of the evaluation HiFi ER 20 hearing protectors raised two hypotheses; we considered that few participants reported full-time use during presentations. Firstly, pop-rock musicians resist using earplug type hearing protectors, because they affect some aspects of music quality or their visual aspect. Secondly, this type of hearing protector, although providing uniform attenuation, is not the most recommended model for this profession. Therefore, it may be interesting to use custom hearing protectors to assure comfort and esthetics, despite a higher cost; it is also possible to adapt different attenuation filters.

A recent study of opera and classical music musicians revealed that 82.6% of the sample already knew about custom hearing protectors; this was the most frequently used model among this group, especially among percussionists.^32^

It is worth noting that not using hearing protectors may contribute significantly to the onset of hearing loss. A study of 42 non-professional pop-rock musicians compared audiological results of musicians that used and did not use hearing protectors; controls were a group of 20 normal hearing subjects. Differences were statistically significant in a comparison of audiometric means between musicians that did not use hearing protectors (8.2 dB) and those that did (2.4 dB). The authors concluded that hearing loss in musicians that used hearing protectors was very similar to the control group. Hearing loss, however, was significantly more pronounced in those who never used hearing protectors. Thus, musicians that did not use hearing protectors were at a higher risk of developing hearing loss.^24^

Further studies with pop-rock musicians could evaluate acceptance of personal hearing protectors with custom filters or individual monitors to ascertain which would be the most appropriate model for this style of music.

As suggested in other studies,^6^^,^^14^^,^^15^^,^^20^^,^^25^ choice of the most appropriate hearing protector according to the requirements of each musician and audiological monitoring may go hand in hand with educational activities for this population, at first to raise awareness about the risks of exposure to noise, as they are used to high noise levels. Suggested preventive measures are: hearing protectors with uniform attenuation or individual monitors, acoustic treatment of the work environment, staying away from loudspeakers, rest intervals, and otoprotective substances, which remain experimental.

Regarding the use of hearing protectors, musicians need to be re-educated by motivation and training, first to adjust for the presence of an object in the ear canal, and second to listen to music and instruments with properly placed hearing protectors.

There is also the possibility of acoustic treatment of venues. This may be difficult to achieve in the short term due to the high costs of such modifications and the fact that bands do not present at only one place. Moreover, even if environmental issues are dealt with, they are limited because the sound source (instrument) is close to the musician, especially in the case of drummers.

Simple measures may be adopted, such as placing the stage as further away as possible from loudspeakers to decrease the level of noise exposure (depending on how speakers are calibrated). Return speakers may be placed close to musicians, and rest intervals during shows may provide periods of auditory rest.^36^

For such changes to happen, it would be necessary to know the particularities of each musical style and to create educational activities to foster cultural and behavioral changes in professional musicians, since hearing is their primary instrument.

Finally, regulations for safe levels of noise exposure among musicians would be appropriate, together with recommended effective preventive measures as a means of establishing safer work practices.

## CONCLUSIONS

• The worst satisfaction evaluation scores about using the HiFi ER 20 hearing protector concerned interference by the protector of high frequency sound perception and full time use of the hearing protector during shows;
• The most common negative sensations while using the hearing protector were dampened voice and pressure in the ears;
• There was a positive correlation between the presence of hearing complaints and the sensation of decreased tinnitus after using the hearing protector;
• 73.9% of musicians scored over 7 to reflect their satisfaction with using the HiFi ER 20 hearing protector, which suggests a favorable tendency towards accepting this device.
